# Personalized Prechemotherapy Education Reduces Peri-Chemotherapy Anxiety in Colorectal Cancer Patients

**DOI:** 10.1155/2021/6662938

**Published:** 2021-03-18

**Authors:** Shasha Li, Lihong Li, Xin Shi, Mingshu Wang, Xiaoli Song, Feng Cui

**Affiliations:** ^1^Department of Oncology, 2nd Affiliated Hospital of Harbin Medical University, No. 246 Xuefu Rd, Harbin 150086, China; ^2^Department of Urology, 2nd Affiliated Hospital of Harbin Medical University, China

## Abstract

**Objective:**

To evaluate the effect of personalized prechemotherapy education in the reduction of peri-chemotherapy anxiety in patients with colorectal cancer.

**Methods:**

Patients admitted to the Department of Oncology with a diagnosis of stage III or IV colorectal cancer and scheduled for initial chemotherapy from January 1, 2017, to June 30, 2019, were retrieved. Patients in the educated group completed the GAD-7 form to evaluate their anxiety level at admission and 14 days after personalized prechemotherapy education, the educator team of which included both physician and nurse staff. Patients in the control group only completed GAD-7 forms at admission and 14 days thereafter without personalized education.

**Results:**

Three hundred and sixty-four patients were enrolled for analysis, including 127 patients who received personalized prechemotherapy education and 237 patients who did not receive education. There were no significant differences in age, gender, education level, or pretreatment GAD-7 scores between the two groups, but significantly lower posttreatment GAD-7 score, and fewer medium to severe posttreatment anxiety patients in the educated group.

**Conclusion:**

Personalized prechemotherapy education involving physician for medical treatment and nursing staff for peri-treatment care, in contrast to traditional brief discussion with physicians during clinic visits and unified informed consent before treatments, may reduce peri-chemotherapy anxiety more efficiently.

## 1. Introduction

According to data from 2012 to 2016, the new cases of colorectal cancer (CRC) were 38.6 per 100,000 men and women per year [1]. The overall percent of CRC patients surviving 5 years is 64%, ranging from an early stage of 90% to a late stage of 14% [2]. While the incidence rate of CRC had been dropping by 3.6% each year from 2007 to 2016 in population aged 55 and above, it rose by 2% each year in the younger population [2]. The prolonged 5-year survival and extended younger age of incidence make it important to keep the surviving CRC patients healthy both physically and psychologically.

Anxiety is a commonly seen psychological response to stressful situations including diseases. The existence of peri-treatment anxiety in CRC patients receiving surgeries and/or chemotherapies and the influence of peri-treatment anxiety on life quality, as well as factors in the development of peri-treatment anxiety have been shown [3].

Chemotherapy is a major treatment for patients with advanced colorectal cancer [4]. However, only one study showed reduction of peri-treatment anxiety by pretreatment education with a controlled design, which mixed different treatment methods and patients with different stages of CRC [5]. In the present study, we evaluated the effect of personalized prechemotherapy education in the reduction of peri-chemotherapy anxiety in late stage CRC patients.

## 2. Methods

The study protocol was approved by the institutional ethical committee. Informed consent for anonymous participation in this study and future publication was obtained from patients.

Starting from January 1st, 2017, our institution has been advocating for personalized patient education. However, there were still quite a lot of admitted patients who did not receive such education due to the availability of clinical staff. Patients admitted to the Department of Oncology with a diagnosis of CRC [6] and scheduled for initial chemotherapy from December 1, 2017, to November 30, 2019, were retrieved. Exclusion criteria were cases received any previous chemotherapy treatment or cases complicated with other cancers or cases with clinically diagnosed psychological problems before the diagnosis of CRC or concurrent medications such as benzodiazepines, serotonin reuptake inhibitors, or other antidepressants or neuropathic pain medication.

The Chinese version of generalized anxiety disorder 7-item scale (GAD-7) [7] was employed to evaluate patients' anxiety level at admission and 14 days after personalized prechemotherapy education during follow-up visits. Individual personalized prechemotherapy education was given by a team consisted of both physician and nurse staff and covered the development, diagnosis, treatment choices, and prognosis of CRC in plain language, as well as the prechemotherapy preparation and postchemotherapy care based on previous studies [8–10]. Useful experience from anonymous previous patients who received similar chemotherapy was discussed. Patients' specific concerns were also discussed during the education session. Patients in the control group completed GAD-7 forms at admission and the 14 days follow-up visits but did not receive dedicated personalized prechemotherapy education. Patients in both groups were free to discuss their disease with physicians during clinic visits and after admission and had informed consent about their treatment plans.

### 2.1. Statistics

Categorical data were expressed as number of cases and analyzed using *χ*^2^-test. Continuous data were shown as mean ± standard deviation (SD) and were analyzed using Student's *t*-test. Prediction equations for preeducational and postchemotherapy anxiety were obtained using logistic regression. SPSS24.0 (IBM Corp, Armonk, NY) was used for statistical analysis. A two-tailed *p* < 0.05 was considered significantly different.

## 3. Results

**Figure 1:**
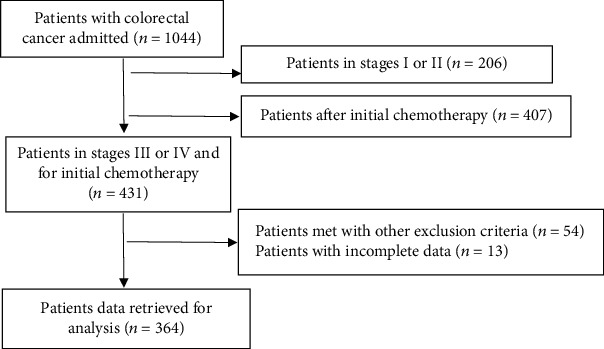
Flow chart of the present study.

There were 1044 patients admitted to the Department of Oncology due to CRC during the study period, and complete data were available from 364 patients who met the inclusion criteria (Figure 1). One hundred and twenty-seven CRC patients received personalized prechemotherapy education, while the rest 237 patients did not. There were no significant differences in age, gender, or education level between the two groups (Table 1, *p* > 0.05). There were no differences in preeducational GAD-7 score or the number of medium to severe anxiety (GAD-7 score above 9) patients before education between the prechemotherapy educated and noneducated groups (Table 1, *p* > 0.05), but significantly lower post-chemotherapy GAD-7 score, and fewer medium to severe anxiety patients after chemotherapy in the prechemotherapy educated group (Table 1, *p* < 0.01). There was a significant reduction in postchemotherapy GAD-7 score in the prechemotherapy educated group (Table 1, *p* < 0.01).

A prediction equation for preeducational anxiety was obtained using logistic regression based on the gender, age, and educational level of all 364 patients, with gender and education level showing significant contribution (*p* <0.05 for both):

(1) $$\begin{array}{c} \log\left[\frac{p}{\left(1-p\right)}\right]=0.537+0.338\text{ gender}-0.11\text{ age}+0.45\text{ education level}, \end{array}$$

where *p* is the probability of having moderate to severe anxiety, gender = 1 if male and =2 if female, and education level =2 if lower than secondary education and =1 if equal to or above secondary education.

A prediction equation for postchemotherapy anxiety was obtained using logistic regression based on the gender, age, and educational level of the 127 patients who received prechemotherapy education, with education level showing significant contribution (*p* < 0.05):

(2) $$\begin{array}{c} \log\left[\frac{p}{\left(1-p\right)}\right]=0.26+0.422\text{ gender}–0.042\text{ age}+0.303\text{ education level}, \end{array}$$

where *p* is the probability of having moderate to severe anxiety, gender =1 if male and =2 if female, and education level = 2 if lower than secondary education and =1 if equal to or above secondary education.

Another prediction equation for postchemotherapy anxiety was obtained using logistic regression based on the gender, age, and educational level of the 237 patients who did not receive prechemotherapy education:

(3) $$\begin{array}{c} \log\left[\frac{p}{\left(1-p\right)}\right]=1.094+0.132\text{ gender}–0.018\text{ age}+0.015\text{ education level}, \end{array}$$

where *p* is the probability of having moderate to severe anxiety, gender =1 if male and =2 if female, and education level =2 if lower than secondary education and =1 if equal to or above secondary education.

## 4. Discussion

**Figure 2:**
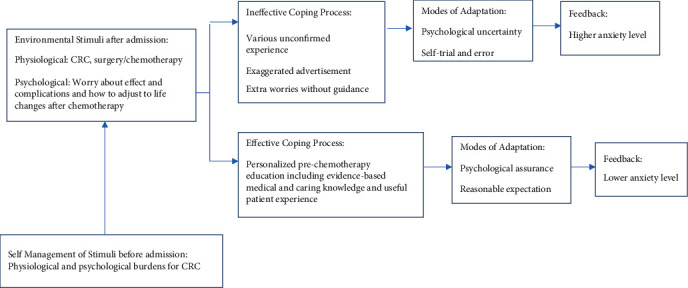
A revised Roy Adaptation Model in the present study.

Anxiety in CRC patients concerning treatment might arises from treatment side effects, treatment costs, time away from family, time away from work, and transportation to treatment sites [11]. Therefore, the provision of comprehensive, useful, timely, and personalized information to CRC patients is needed [12]. Nowadays, all kinds of information related to CRC therapies may come from different sources. Some may even be exaggerated advertisements. Others may be experience from specific cases that are not suitable for the general CRC population. A comprehensive personalized prechemotherapy education by doctors and nurses may systematically improve the overview of CRC patients about how to deal with those potential problems, set realistic expectations, and be prepared for potential chemotherapy and postchemotherapy issues. It has been shown that anxiety begins at treatment planning and increases to high level on admission to hospital [13]. According to the modified Roy Adaptation Model in Figure 2 [14], more effort is needed in the pre- or early hospitalization of CRC patients to minimize anxiety level as well as the potential need for extra medical and care resources [15].

The involvement of nursing staff in personalized prechemotherapy patient education to reduce patients' anxiety has not been adequately reported. A literature search on February 20, 2020, using the key words of “colon rectal cancer anxiety” in PubMed produced 654 papers, whereas addition of extra key words of “nurse OR nursing” only produced 94 papers. Concerning CRC patients receiving chemotherapy, some studies have reported pretreatment education and follow-up without control groups or details of education [16], some employed web-based education with small number of patients without controls [17], some used tailored information pack according to the treatment plan [5], and some had telephone sessions nearing or after the completion of chemotherapy [18–21]. However, conversation in person might be the best option to reduce anxiety [22]. In our present study, in collaboration with physicians who concentrated on medical aspect, the nursing staff who gave instructions on chemotherapy and postchemotherapy care greatly benefited the CRC patients and effectively reduced peri-chemotherapy anxiety.

Generalized anxiety disorder (GAD) is among the most common anxiety disorders in general medical practice as well as in the general population [23]. The GAD-7 form was among the most popular scales for the measurement of GAD [24]. Other scales have been used to measure anxiety, including Hospital Anxiety and Depression Scale (HADS) [5, 16, 25]. The HADS has been used to identify both anxiety and depression in nonpsychiatric clinics and contains an Anxiety (HADS-A) and a Depression subscale (HADS-D). It has been shown that both GAD-7 and HADS-A showed AUC of adequate diagnostic accuracy and hence are applicable for GAD screening in cancer patients [26].

There are potential personal factors contributing to the level of anxiety, such as gender [23] or education level [27]. In this study, we found significant contribution of gender of female and low education level to prechemotherapy anxiety but not age. CRC patients are generally older (the mean age was 68.36 years old in our study) and are more likely to miss the details of chemotherapy by brief discussions with physicians. A personalized prechemotherapy education session might be helpful for those patients to comprehensively understand and systematically memorize the chemotherapy procedures and care tips so that anxiety levels could be reduced [28].

## 5. Conclusions

This study was the first one to explore the effect of personalized prechemotherapy education in reducing peri-chemotherapy anxiety in CRC patients. Personalized prechemotherapy education provided in collaboration by physician and nursing staff, as well valuable experience from previous patients was beneficial in reducing peri-chemotherapy anxiety in CRC patients. Education level has an important role in the development and severity of peri-chemotherapy anxiety.

## Figures and Tables

**Table 1:** Clinical characteristics of enrolled patients.

Characteristics	Prechemotherapy educated (127 cases)	Not educated (237 cases)	*p* value (between groups)	Relative risk	95% CI
Age^&^	69.28 ± 6.68	68.09 ± 6.92	0.12^∗^	—	—
Male^$^	84	145	0.74^∗∗^	1.08	(0.92, 1.27)
Education level (≥ secondary education)^$^	46	68	0.14^∗∗^	0.89	(0.77, 1.04)
Prechemotherapy GAD-7^&^	9.88 ± 3.01	10.07 ± 2.70	0.55^∗^	—	—
Moderate to severe anxiety^$^	58	128	0.13^∗∗^	0.85	(0.68, 1.06)
Postchemotherapy GAD-7^&^	6.85 ± 2.90	9.70 ± 2.16	<0.01^∗^	—	—
Moderate to severe anxiety^$^	26	121	<0.01^∗∗^	0.40	(0.28, 0.58)
*p* value (before vs. after education)	<0.01^∗^	0.10^∗^	—	—	—
Change in GAD-7^&^	−2.19 ± 1.60	−1.43 ± 0.95	<0.01^∗^	—	—

^$^Number of patients. ^&^Mean ± SD. ^∗^*t*-test. ^∗∗^*χ*^2^ test.

## Data Availability

The data used to support the findings of this study are available from the corresponding author upon request.
